# Supplementary material for: Genetic variation, environment and demography intersect to shape Arabidopsis defense metabolite variation across Europe
Source: eLife. 2021 May 5;10:e67784. doi: 10.7554/eLife.67784 (PMC8205490; doi:10.7554/eLife.67784)
Supplement: Figure 4—source data 1. — Linear model for MAM status (carbon side chain length) was conducted with the indicated environmental parameters, for the northern and southern collection, separately (for more details, see Methods). The tables show p values for each term from the linear model. For the interaction with geography, the linear model was run using the total dataset, and the geography parameter (north or south) was added to the model. [file elife-67784-fig4-data1.pptx]

## Slide 1
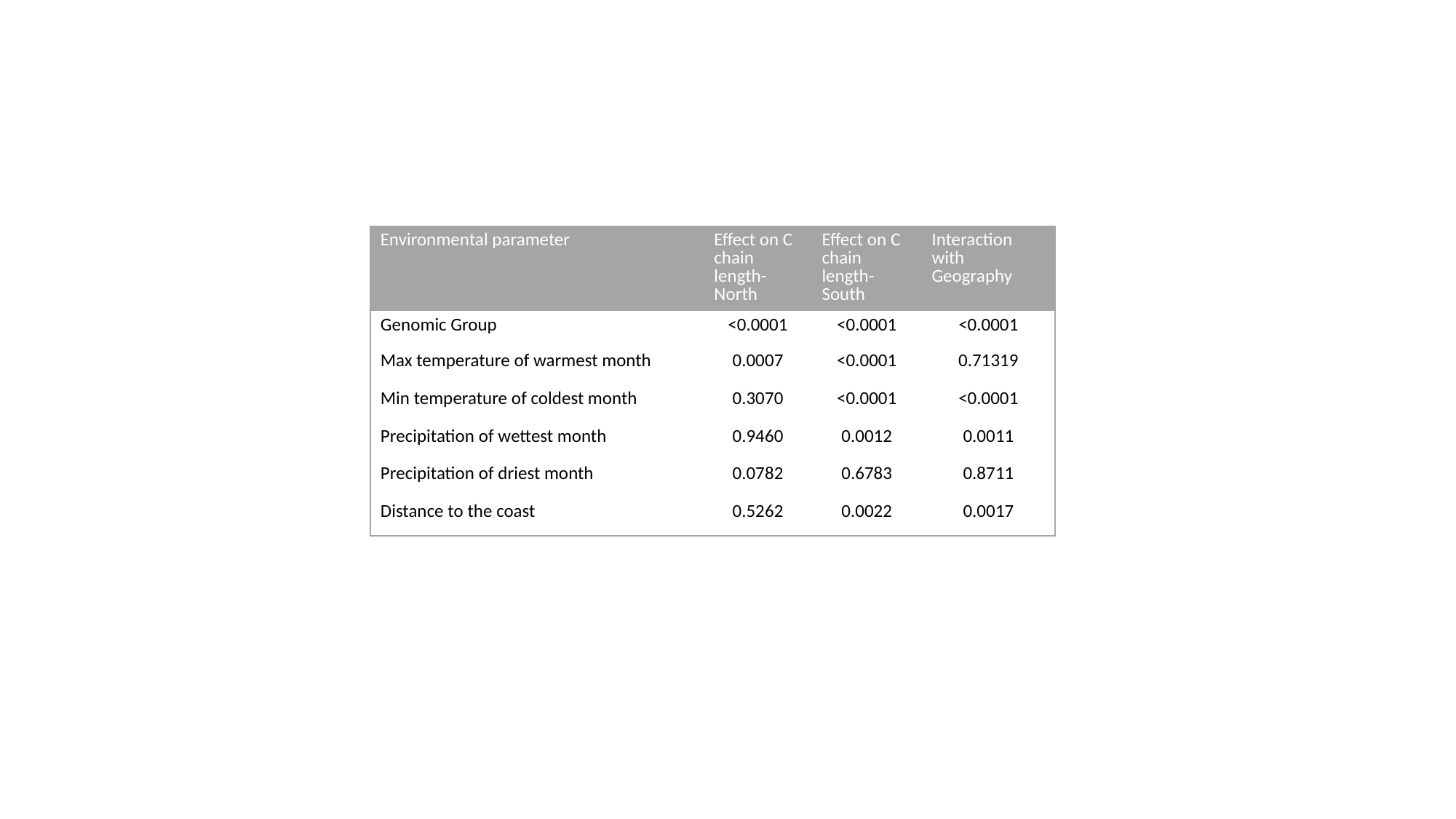

| Environmental parameter | Effect on C chain length- North | Effect on C chain length- South | Interaction with Geography |
| --- | --- | --- | --- |
| Genomic Group | <0.0001 | <0.0001 | <0.0001 |
| Max temperature of warmest month | 0.0007 | <0.0001 | 0.71319 |
| Min temperature of coldest month | 0.3070 | <0.0001 | <0.0001 |
| Precipitation of wettest month | 0.9460 | 0.0012 | 0.0011 |
| Precipitation of driest month | 0.0782 | 0.6783 | 0.8711 |
| Distance to the coast | 0.5262 | 0.0022 | 0.0017 |
